# Environmental factors affect the communities of avian *Plasmodium* in two species of *Culex* mosquitoes

**DOI:** 10.1038/s41598-026-51361-w

**Published:** 2026-05-05

**Authors:** Ana Irina Martín-López, María José Ruiz-López, Josué Martínez-de la Puente, Sergio Magallanes, Shirin Taheri, Santiago Ruiz, Jordi Figuerola

**Affiliations:** 1 Department of Conservation Biology and Global Change, Estación Biológica de Doñana, CSIC, Calle Américo Vespucio, 26, Seville, 41092 Spain; 2 Servicio de Control de Plagas de la Diputación de Huelva, Huelva, Spain; 3 CIBER de Epidemiología y Salud Pública (CIBERESP), Avenida Monforte de Lemos 3-5, Madrid, 28029 Spain

**Keywords:** Avian malaria, β-diversity, *Culex perexiguus*, *Culex pipiens*, Environmental factors, Vector-borne pathogens, Diseases, Ecology, Ecology, Zoology

## Abstract

Understanding vector-borne pathogen transmission is a priority due to its impact on human and animal health. While mosquitoes are key hosts of avian malaria, most studies focus on birds. This study examines how environmental factors influence *Plasmodium* communities in *Culex pipiens* and *Culex perexiguus* and compares lineage composition and spatial patterns. Mosquitoes were collected from 16 sites in southwest Spain over two years. *Plasmodium* detection was performed using nested PCR, and lineages were identified by SANGER sequencing. Linear mixed models tested environmental effects on *Plasmodium* communities, and correspondence analysis and Mantel tests explored interspecific and spatial relationships. In *Cx. pipiens*, *Plasmodium* prevalence increased with NDVI, while lineage richness declined with land surface temperature. In *Cx. perexiguus*, prevalence and lineage richness increased with minimum temperature, and diversity decreased with land surface temperature. *Plasmodium* communities differed between mosquito species, with community similarity correlating with geographical distance only in *Cx. pipiens*. These findings suggest that environmental factors shape *Plasmodium* communities in mosquitoes, and that vector identity influences lineage structure and distribution. Integrating vector ecology and environmental context is essential to better understand avian malaria transmission dynamics in changing environments.

**Supplementary Information:**

The online version contains supplementary material available at 10.1038/s41598-026-51361-w.

## Introduction

Understanding the ecology and evolution of vector-borne diseases is a priority because they threaten animal and human health. These diseases result in more than 700,000 human fatalities annually, affecting 300 million individuals and putting 6.3 billion people at risk^1^. Human malaria is one of the most important vector-borne diseases, having caused over 150 to 300 million deaths in the last century^2^. This vector-borne disease is caused by a protozoan parasite of the genus *Plasmodium* (order Haemosporida). Malaria parasites affect not only mammals, but also other vertebrates, such as birds and reptiles^3^. These parasites have similar transmission cycles but differ in their vertebrate hosts and mosquito vectors. For example, while *Anopheles* are the vector of human malaria parasites, mosquitoes of the *Culex* genus may play a central role in the transmission of avian *Plasmodium*^3^.

Both intrinsic and extrinsic factors determine the transmission dynamics of avian malaria. Intrinsic factors include those related to both vertebrate hosts and insect vectors, such as the host species and sex, age and physiology, among others^4^. For instance, sex-specific differences in immunocompetence and resource allocation can lead to differential infection risk in great tits, with males exhibiting reduced cellular immune responses and greater parasite-associated reductions in body condition compared to females^5^. Furthermore, extrinsic factors including abiotic factors such as environmental conditions and biotic factors such as host/vector density or host/vector population structure may largely determine the transmission dynamics of avian *Plasmodium* in the wild^6^. It is suggested that species-rich communities harbor a lower fraction of competent hosts, reducing pathogen transmission success and, consequently, pathogen prevalence, producing a dilution effect^7^.

Previous studies highlighted the importance of environmental factors in the composition of *Plasmodium* communities in birds, affecting their prevalence, lineage richness and diversity depending on local habitat characteristics and the host species studied^8,9^. In birds, proximity to water resources is one of the most important predictors of *Plasmodium* prevalence and richness^10^. Small water bodies with stagnant water provide suitable habitats for mosquito breeding, supporting mosquito abundance^11^ and increasing the risk of vector-borne parasite infections in birds^12^. Higher values of Normalized Difference Vegetation Index (NDVI) have also been associated with higher *Plasmodium* prevalence and richness in birds^10^. Additionally, higher temperatures and lower precipitation lead to greater *Plasmodium* prevalence and richness in birds^13^. However, this depends on the altitude of the studied localities, which causes spatial variations in parasite prevalence and richness associated with temperature and precipitation gradients^9^. Finally, the prevalence, richness, and diversity of *Plasmodium* in birds vary spatially and temporally depending on the habitat where the host species is found^14^.

However, *Plasmodium* life cycle involves transmission and development in mosquitos and vertebrates, two very different organisms that will impose very different selection pressures on *Plasmodium*^3^. In contrast to studies on birds, current knowledge of the environmental factors determining avian *Plasmodium* infection patterns in mosquitoes is limited^15^. Studies conducted to date report a positive correlation between the prevalence of *Plasmodium* in *Culex pipiens* and the distance to saltmarshes and rivers, with a higher *Plasmodium* richness in mosquitoes in natural areas compared to those of rural and urbanized areas^16^. Furthermore, NDVI and both ambient temperature and land surface temperature may affect parasite prevalence, richness and diversity by influencing mosquito abundance^17^. Although humidity and water balance are crucial for mosquito activity patterns^18^, the potential influence of factors such as vapor pressure or evapotranspiration on *Plasmodium* prevalence and diversity in mosquitoes remains poorly known^19^. Because arthropod vectors are ectothermic, climate change and other drivers of global change may increase the incidence of avian *Plasmodium*, at least, in temperate areas^20^. Thus, it is key to understand how environmental factors affect the interaction between wildlife malaria parasites and mosquito vectors in the wild.

Overall, these studies highlight the complex dynamics of avian *Plasmodium* in the wild and the need to understand how environmental factors affect them. Considering the limited information available for mosquito vectors^15^, we aim to understand which environmental factors including climate and landscape predict the prevalence, richness, and diversity of *Plasmodium* in two mosquito species. We hypothesize that abiotic factors, particularly NDVI and temperature, will predict the composition, prevalence, richness and diversity of avian *Plasmodium* communities in both mosquito species. These variables have been consistently associated with host bird prevalence and community composition in previous studies^10,13^. They may also directly affect mosquito abundance and survival, as well as pathogen development^17^. In addition, we aim to compare the *Plasmodium* lineage communities in both mosquito species and to test spatial and temporal patterns in the structure of the community. We predict that the *Plasmodium* communities between mosquitoes will differ due to their distinct intrinsic characteristics, such as differences in their host feeding patterns and habitat preferences, that may expose them to different host and pathogen lineages^21,11^. We focus this study on two mosquito species, namely *Cx. pipiens* and *Cx. perexiguus*, due to their relevance in the transmission of avian *Plasmodium* under natural conditions in the study area^22^. To address these objectives, we sampled mosquitoes from 16 sites in southwest Spain over two years and screened them for *Plasmodium* infection using nested PCR and Sanger sequencing to identify parasite lineages. Then, we used linear mixed models to evaluate the influence of environmental variables on parasite prevalence, richness and diversity, and multivariate analyses (CA and Mantel test) to compare *Plasmodium* community composition in both mosquito species.

## Results

### Variation in *Plasmodium* lineages and prevalence among mosquito species

We analyzed 109,460 female mosquitoes (18,316 *Cx. pipiens* and 91,144 *Cx. perexiguus)* grouped in 3633 mosquito pools, corresponding to 2395 pools of *Cx. perexiguus* and 1238 pools of *Cx. pipiens.* The overall prevalence of avian *Plasmodium* in mosquitoes was higher in *Cx. pipiens* (mean: 1.33%; CI 95%: 1.16, 1.53; 218 of 1238 pools) than in *Cx. perexiguus* (mean: 0.84%; CI 95%: 0.81, 0.94; 668 of 2395 pools) (Est = 0.180 ± 0.030, t = 6.001, df = 44.959, *p* < 0.001). In *Cx. pipiens*, 14 of 16 localities presented, at least, one positive mosquito pool (see Table S1). In *Cx. perexiguus*, 10 localities had, at least, one positive mosquito pool (see Table S2).

**Fig. 1:**
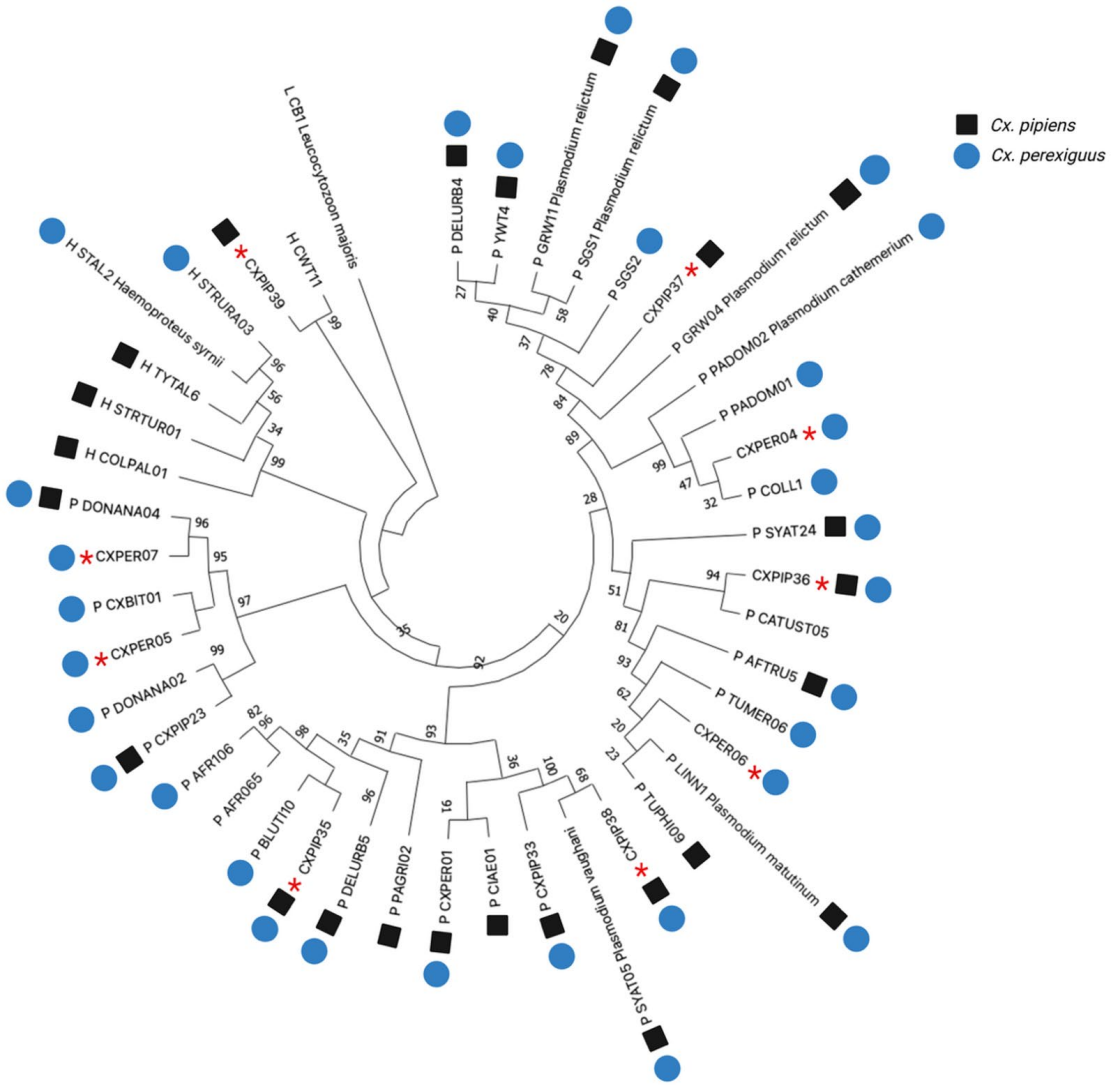
Phylogenetic tree including all *Plasmodium* lineages found in this study (see Table S3) and their corresponding morphospecies, as indicated when available on the MalAvi database^27^. New lineages identified for the first time in this study are marked with a red asterisk (*). To better illustrate the phylogenetic relationships, we also included the most closely related *Plasmodium* lineages according to MalAvi^27^, regardless of whether they were found in our study. Lineages found in *Culex pipiens* are marked with blue squares, while those found in *Culex perexiguus* are marked with black circles. Lineages without these symbols were not detected in this study but are included for phylogenetic context.

We determined the identity of *Plasmodium* lineages in 73.49% and 77.64% of the positive *Cx. pipiens* and *Cx. perexiguus* pools, respectively. 13 samples showed evidence of mixed infections by different *Plasmodium* lineages. Overall, we found 35 different lineages. Of those, 17 lineages were present in both mosquito species, 5 lineages were found only in *Cx. pipiens*, and 13 only in *Cx. perexiguus* (Fig. 1). *Cx. pipiens* tended to show lower *Plasmodium* richness than *Cx. perexiguus* (Est = -2.231 ± 1.175, t = -1.900, df = 31.696, *p* = 0.067), but we did not find significant differences in *Plasmodium* diversity between the two mosquito species (Est = -0.027 ± 0.184, t = -0.146, df = 32.618, *p* = 0.885). We identified 9 new lineages (GenBank accession no PQ798937- PQ798945; see Table S3): 2 were found only in *Cx. pipiens*, 4 in *Cx. perexiguus*, and 3 in both species. The most abundant lineages in *Cx. pipiens* were SYAT05 (36.08%), LINN1 (14.56%), SGS1 (12.66%) and CXPIP33 (5.06%). SYAT05 was found in *Cx. pipiens* in 11 different localities. In *Cx. perexiguus* the most abundant lineages were LINN1 (35.41%), SYAT05 (28.79%), CXPIP36 (6.23%) and AFTRU5 (5.45%). LINN1 was found in *Cx. perexiguus* from 7 different localities.

### Environmental effects on *Plasmodium* prevalence, richness and diversity

We found that the most supported models, according to AIC, were those explained by a single environmental variable among all those tested, for both *Cx. pipiens* and *Cx. perexiguus* (Table 1). *Plasmodium* prevalence in the two mosquito species was significantly associated with different environmental variables. In *Cx. pipiens*, *Plasmodium* prevalence was positively associated with NDVI, while in *Cx. perexiguus* was positively associated with the minimum temperature (Table 2). *Plasmodium* richness in *Cx. pipiens* was negatively related to land surface temperature, whereas in *Cx. perexiguus*, it was positively associated with the minimum temperature (Table 2). We did not find any significant relationship between *Plasmodium* diversity in *Cx. pipiens* and any of the environmental variables tested. However, *Plasmodium* diversity was negatively related to land surface temperature in *Cx. perexiguus* (Table 2).

**Table 1:** Models testing the relationship between environmental factors and *Plasmodium* prevalence (a, d), richness (b, e) and diversity (c, f) in *Culex pipiens* (a-c) and *Culex perexiguus* (d-f) measured for a buffer of 2 km. Only models within ∆AICc < 2 of the best-supported model are shown; models with ∆AICc > 2 are not presented. NDVI = normalized difference vegetation index, Tmin = minimum temperature, LST = land surface temperature, Tmean = mean temperature, K = number of model parameters, ∆AICc = difference in AIC compared to the best model, W_i_ = Akaikes weight, log(l) = log-likelihood value.

Model	K	∆AICc	W_i_	log(l)
a) *Plasmodium* prevalence in *Culex pipiens* (*n* = 29)				
NDVI + Tmin + 1/Loc + 1/Year	5	0.00	0.69	28.12
NDVI + 1/Loc + 1/Year	4	1.20	0.34	29.21
b) *Plasmodium* richness in *Culex pipiens* (*n* = 29)				
LST + Tmean + 1/Loc + 1/Year	5	0.00	0.48	− 35.53
LST + 1/Loc + 1/Year	4	0.70	0.35	− 35.08
c) *Plasmodium* diversity in *Culex pipiens* (*n* = 24)				
1 + 1/Loc + 1/Year	3	0.00	0.44	− 16.93
Tmean + Vapor pressure + 1/Loc + 1/Year	5	1.10	0.26	− 14.12
Tmean + 1/Loc + 1/Year	4	2.00	0.17	− 16.41
d) *Plasmodium* prevalence in *Culex perexiguus* (*n* = 20)				
Tmin + 1/Loc + 1/Year	4	0.00	0.81	14.38
e) *Plasmodium* richness in *Culex perexiguus* (*n* = 20)				
Tmin + 1/Loc + 1/Year	4	0.00	0.23	− 24.97
f) *Plasmodium* diversity in *Culex perexiguus* (*n* = 18)				
LST + 1/Loc + 1/Year	4	0.00	0.91	− 5.07

**Table 2:** Estimate (Est), standard error (SE), t-values (t), degrees of freedom (df), p-values (p) and R^2^ from the LMMs with the lowest AIC analyzing the relationship between different environmental factors and *Plasmodium* prevalence, lineage richness and diversity in *Culex pipiens* and *Culex perexiguus*. Significant p-values (*p* < 0.05) are marked in bold. NDVI = normalized difference vegetation index, LST = land surface temperature, Tmean = mean temperature, Tmin = minimum temperature, Tmax = maximum temperature.

*Culex pipiens*					*Culex perexiguus*			
	Est (± SE)	t	df	*p*	Est (± SE)	t	df	*p*
a)*Plasmodium* prevalence								
Intercept	− 0.05 (± 0.01)	3.00	11.11	**0.03**	− 0.07 (± 0.01)	3.53	7.14	**0.009**
NDVI	0.05 (± 0.01)	2.96	17.50	**0.008**	–	–	–	–
Tmin	–	–	–	–	0.08 (± 0.02)	3.91	8.82	**0.004**
R^2^	0.67				0.51			
b) *Plasmodium* richness								
Intercept	− 3.31 (± 1.23)	2.69	1.07	**0.21**	9.00 (± 1.95)	4.61	3.87	**0.01**
LST	− 1.13 (± 0.45)	− 2.46	7.89	**0.03**	–	–	–	–
Tmin	–	–	–	–	4.65 (± 0.98)	4.70	4.14	**0.008**
R^2^	0.65				0.82			
c) *Plasmodium* diversity								
Intercept	0.83 (± 0.29)	2.77	0.45	0.40	1.32 (± 0.23)	5.69	0.97	**0.11**
Tmean	0.17 (± 0.20)	0.85	2.89	0.45	–	–	–	–
LST	–	–	–	–	− 0.61 (± 0.11)	− 5.67	7.18	**< 0.001**
R^2^	0.07				0.83			

### Characterization of *Plasmodium* communities

The correspondence analysis (CA) for mosquito species did not clearly separate localities or years based on specific lineage prevalences (Fig. S1). The first axis of the correspondence analysis (CA) explained 65.9% of the variance and the second axis explained 19.4% of the variance. We found significant differences between mosquito species on the second axis of the CA (F_1,36_ = 4.737, *p* = 0.03) but not in the first axis (F_1,36_ = 1.198, *p* = 0.28).

When analyzing the β-diversity, Mantel tests carried out using either Jaccard or Bray-Curtis metrics, yielded similar qualitative results. Therefore, we report only the results based on Bray-Curtis. There was no significant relationship between the β-diversity of *Plasmodium* in *Cx. pipiens* and *Cx. perexiguus* (*r* = 0.22, *p* = 0.19). β-diversity of *Plasmodium* in *Cx. pipiens* was significantly related to geographic distance (*r* = 0.67, *p* = 0.03), but no significant relationship was found between β-diversity of *Plasmodium* in *Cx. perexiguus* matrix and geographic distance (*r* = 0.29, *p* = 0.14), even after controlling for environmental relatedness in a partial mantel test (*r* = 0.69, *p* = 0.03 with *Cx. pipiens*; *r* = 0.29, *p* = 0.15 with *Cx. perexiguus*). However, environmental relatedness did not show a significant correlation with β-diversity of *Plasmodium* for either *Cx. pipiens* (*r* = -0.16, *p* = 0.82), or *Cx. perexiguus* (*r* = 0.02, *p* = 0.47), even after controlling for geographic distance in a partial mantel test (*r* = -0.23, *p* = 0.90 with *Cx. pipiens*; *r* = 0.02, *p* = 0.40 with *Cx. perexiguus*).

## Discussion

Studies of avian malaria have primarily focused on the relationships between bird hosts and their parasites, often overlooking the role of vectors in transmission dynamics. In this study, we investigated the environmental and ecological factors influencing the prevalence and diversity of *Plasmodium* lineages in two mosquito species, *Cx. pipiens* and *Cx. perexiguus*, in southwestern Spain. Our results suggest that vector identity is a key component influencing the structure of the *Plasmodium* community, as shown by the differences observed between *Cx. pipiens* and *Cx. perexiguus*. Although *Plasmodium* lineage richness and diversity did not differ between the two vectors, the higher prevalence in *Cx. pipiens* and the associations with different environmental factors suggest that each vector might contribute differently to parasite circulation. These findings highlight the key role of environmental conditions and vector ecology in shaping *Plasmodium* community structure and distribution, emphasizing the importance of integrating environmental and vector factors to better understand avian malaria transmission dynamics.

The average prevalence of *Plasmodium* for both *Cx. pipiens* and *Cx. perexiguus* was within the range reported by previous studies in the Iberian peninsula^16^. The higher *Plasmodium* prevalence observed in *Cx. pipiens* is consistent with previous evidence that identifies this species as a competent and potentially primary vector of avian malaria parasites in the area ^22^. This pattern might reflect differences between species associated with host use, or vector competence and transmission efficiency ^23^. *Cx. pipiens* is known to exhibit flexible feeding behavior ^21^ and broad habitat use ^24^, which may increase its contact with a wide range of avian hosts and enhance transmission opportunities. In contrast, the comparatively lower prevalence in *Cx. perexiguus* may reflect a more specialized ecology ^25^, potentially constraining its role in maintaining high infection levels despite harboring a comparable diversity of *Plasmodium* lineages.

In addition, variation in bird community composition may explain, at least in part, the observed patterns of *Plasmodium* richness and diversity. Although overall *Plasmodium* richness and diversity did not differ significantly between the two mosquito species, richness tended to be higher in *Cx. perexiguus*. In this species, most of the lineages were detected only in five localities that are close to rice fields. Because this is a preferred habitat for *Cx. perexiguus* ^25^, at these sites they were very abundant and there was a higher percentage of infected pools than for *Cx. pipiens*. These areas also present a high abundance and diversity of birds ^26^. SYAT05 and LINN1 were the most abundant lineages in *Cx. pipiens* and *Cx. perexiguus*, although they presented different prevalences. Both lineages are generalists, known to infect a wide range of birds and mosquito vectors ^27^. SYAT05 has been found in seven mosquito species and LINN1 in six, including both *Cx. pipiens* and *Cx. perexiguus* (Vector Data Table in MalAvi, version 2.5.9). In birds, SYAT05 has been recorded in 37 species, including 18 that are present in Spain, while LINN1 in 32 species, including 17 that can be found in Spain (Grand Lineage Summary Table in MalAvi, version 2.5.9). Among these, the common blackbird, *Turdus merula*, is a common host of both SYAT05 and LINN1 *Plasmodium* lineages ^27^. This bird species is abundant in the area and generally shows a high prevalence of *Plasmodium* infection in different habitats ^14^. Additionally, *T. merula* is a preferred host of *Cx. pipiens* mosquitoes ^28^, which may explain the high prevalence of infection found in mosquitoes. Furthermore, approximately 11.2% of the blood meals of *Cx. perexiguus* in southern Spain derived from *T. merula* ^21^. During the 2020 West Nile virus outbreak in South West Spain, *T. merula* presented the highest prevalence of antibodies, suggesting that this species was highly preferred by the main West Nile virus vectors, *Cx. perexiguus* and *Cx. pipiens* ^29^.

Despite sharing dominant *Plasmodium* lineages, we did not find any relationship between *Plasmodium* communities of *Cx. pipiens* and *Cx. perexiguus*. Differences in community composition might be driven by variation in the relative abundance of these shared lineages, as well as the presence of less common, rare lineages. This suggests that even when the same lineages are circulating, their distribution across mosquito species can differ. As observed for prevalence these differences likely reflect species-specific ecological and biological traits, including feeding behavior and vector competence, which influence their roles in parasite transmission ^23^.

In addition, while *Plasmodium* community composition was significantly correlated with geographical distance in *Cx. pipiens*, it was not in *Cx. perexiguus*. *Plasmodium* lineages present isolation-by-distance relationships in both molecular divergence and community similarity in birds ^30^. This implies that geographically closer *Cx. pipiens* populations have more similar *Plasmodium* communities compared to those farther apart. We have observed that the *Cx. pipiens* populations in our study area had a continuous distribution ^24^, which may facilitate gene flow between populations. On the contrary, *Cx. perexiguus* shows a patchier distribution with a high preference for certain habitats such as rice fields ^25^, which may explain the low correlation between *Plasmodium* community composition and geographical distance.

Environmental conditions are important determinants of parasite infection patterns in bird host and vectors. Here, we found a positive association between NDVI and the prevalence of *Plasmodium* in *Cx. pipiens.* These are similar patterns to those found in birds in the study area ^10^. Higher values of NDVI likely reflect increased vegetation cover development which requires availability of water, which may increase the availability of breeding and resting sites for mosquitoes, as well as greater food resources for both mosquitoes and birds ^31^. These conditions may lead to increased mosquito and bird abundance and consequently greater *Plasmodium* prevalence and diversity ^31^. In contrast, although NDVI was evaluated for *Cx. perexiguus*, it was not the variable that best explained variation in prevalence. This difference may reflect ecological distinctions between the two species. We hypothesize that, because *Cx. pipiens* is more generalist ^24^, it may be more influenced by broad environmental gradients such as vegetation greenness captured by NDVI. In contrast, *Cx. perexiguus* is more closely associated with specific habitats such as rice fields ^25^, suggesting a greater degree of specialization. As a result, NDVI may be less relevant for explaining the infection dynamics in *Cx. perexiguus* among other factors because rice-cultivated fields will present similar NDVI values.

We also found that *Plasmodium* prevalence and richness in *Cx. perexiguus* was positively associated with minimum temperature. These results are consistent with those from Pérez-Rodríguez et al. ^32^ who found a positive relationship between minimum temperature and *Plasmodium* prevalence and richness in *Sylvia atricapilla* from Spain. Although the effect of temperature on mosquitoes can vary by population ^33^ and season ^34^, higher temperatures are generally associated with increased mosquito abundance ^35^, favoring pathogen transmission. In contrast, we found that high land surface temperatures were negatively associated with *Plasmodium* richness in *Cx. pipiens* and *Plasmodium* diversity in *Cx. perexiguus*. Although previous studies have also linked higher land surface temperature with an increase in *Cx. pipiens* and *Cx. perexiguus* abundance ^17^, one possible explanation is that the optimal development of different avian *Plasmodium* lineages in different mosquito species may occur under different environmental conditions. This may result in local and temporal differences in the composition of the *Plasmodium* community in mosquitoes ^34^. In this case, high land surface temperature may contribute to lower *Plasmodium* richness and diversity, as certain lineages may be more competitive and abundant ^33^. In addition, it is important to note that *Cx. perexiguus* is mainly an African species that presents its northern distribution limit in southern Europe, while *Cx. pipiens* is a mosquito species widely distributed in Europe, even reaching areas of Scandinavia ^36^. Finally, the absence of a significant relationship between *Plasmodium* diversity in *Cx. pipiens* and environmental variables may reflect the influence of multiple factors on *Plasmodium* lineage transmission, including vector competence ^37^, or interactions with other potential *Plasmodium* vector species present in each studied locality.

While our research provides valuable insights into the environmental factors influencing avian *Plasmodium* transmission, the environmental influences detected were relatively subtle because best-supported models retained only a single predictor. The use of spatially averaged data with limited locality–year observations may have reduced our ability to detect multi-factor effects ^38^. Thus, the variables identified should be interpreted as dominant environmental gradients rather than comprehensive determinants of *Plasmodium* community structure. In addition, while considering a 2 km buffer based on the typical flight range of *Culex* mosquitoes ^39^ is appropriate for our study, it does not account for fine-scale microhabitat conditions, such as ephemeral breeding sites or localized vegetation and irrigation patterns, which could further influence mosquito distribution and infection risk. Finally, additional factors such as biotic factors (i.e. host community or vector community), vector competence and the potential involvement of other local mosquito species (e.g., *Cx. modestus*) are likely to further influence transmission dynamics ^23^. Future work addressing these aspects will refine our understanding of the complex ecological networks driving avian malaria transmission and underscore the importance of integrating vector ecology and environmental factors in studies of parasite transmission.

In conclusion, we present evidence that environmental factors play a key role in avian malaria transmission dynamics, shaping the *Plasmodium* communities present in two mosquito species. Our study highlights the importance of mosquito vectors, which remain understudied despite their relevance for parasite transmission, and reveals that *Plasmodium* community composition varies with vector species and population structure.

## Methods

### Mosquito sampling and identification

**Fig. 2:**
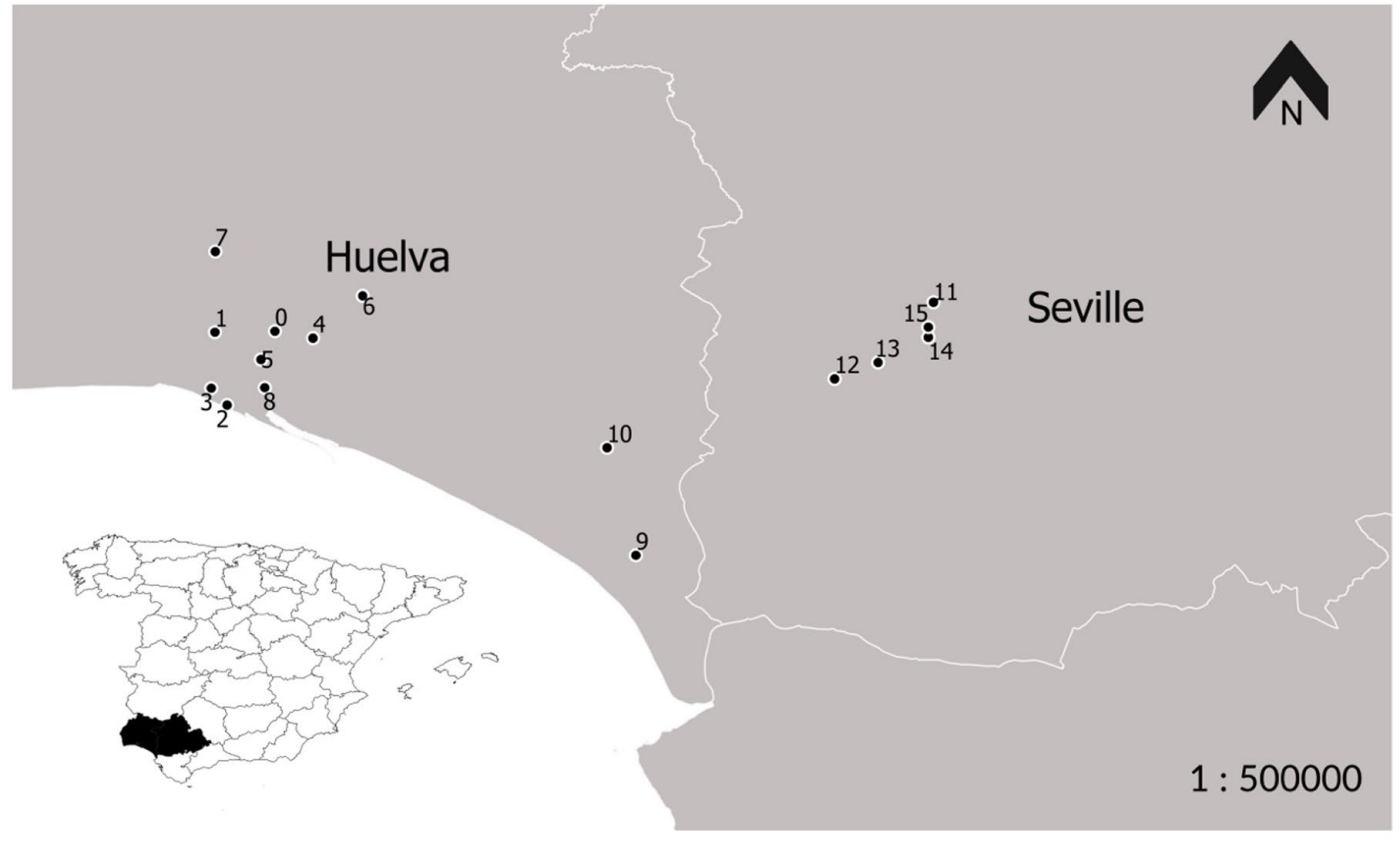
Distribution of the 16 sampling sites in Southwest Spain. Localities are distributed across the provinces of Huelva and Seville in Andalusia. Numbers correspond to the localities: (0) Los Álamos, (1) Corrales, (2) Casa de los Ingleses, (3) Salinas del Astur (Punta Umbría), (4) EDAR Moguer, (5) EDAR Huelva, (6) Granja Escuela, (7) Gibraleón, (8) Celestino Mutis, (9) Palacio de Doñana, (10) El Rocío, (11) Palomares del Río, (12) Dehesa de Abajo, (13) Cañada de los Pájaros, (14) Puebla del Río, (15) Coria del Río.

This study was carried out in Andalusia, an area characterized by a hot summer Mediterranean climate (Csa) ^40,41^. Mosquitoes were captured in 2021 and 2022 from March to November in 16 localities in the provinces of Huelva and Seville (Fig. 2). Trapping was carried out using BG-Sentinel traps (Biogents, Ratisbona, Germany) or CDC incandescent light traps (Center for Disease Control and Prevention, Atlanta, USA), in both cases baited with CO_2_ (dry ice). Three BG traps were active for 24 h, once a week at the sites where the 2020 West Nile Virus (WNV) outbreak occurred ^29^, and once a month in the other localities (see Table S4).

Once in the laboratory, the mosquitoes were separated by date of sampling, locality, sex and classified into species based on their morphology with a stereomicroscope following Moskeytool ^42^. Mosquito females of the same species captured on the same date and locality were grouped in pools of up to 50 females. Mosquitoes grouped in pools did not show any rest of blood meals in their abdomen. Working with pools allows us to maximize the number of mosquitoes analyzed, reducing the cost given the usually low prevalence of infections ^43^. All identification and classification procedures were performed over a chilling table to prevent the degradation of nucleic acids.

### Molecular and phylogenetic analysis of avian *Plasmodium*

Genomic DNA was extracted for each mosquito pool using the Maxwell^®^ 16 LEV system Research (Promega, Madison, WI, USA) extraction robot and the Viral Total Nucleic Acid Purification kit (Promega, Madison, WI, USA) following the manufacturer’s instructions. *Plasmodium* was detected using a nested PCR described by Hellgren et al. ^44^. To reduce the occurrence of false negatives, two replicates of each sample were analyzed, and the samples were considered PCR positive if at least one of the two replicates was positive. PCR-positive samples with only one faint band were repeated in a third PCR replicate to obtain a clean band. The amplicons were initially sequenced unidirectionally using the primer Haem R2 ^44^ by Macrogen (Macrogen Inc., The Netherlands and Spain) or the Genomics Unit at the Complutense University of Madrid. The sequences obtained were analyzed with Geneious Prime software (version 2023.0.4; https://www.geneious.com) ^45^. *Plasmodium* lineages were identified using the Basic Local Alignment Search tool (BLAST) in a specific database for avian haemosporidian parasites, MalAvi, ^27^ and GenBank 46. In accordance with the standard practice adopted in Bensch et al. ^27^, parasite lineages were delimited based on sequence variation in the amplified cyt b fragment, with a single nucleotide substitution considered sufficient to define a distinct lineage. If the resulting sequences matched several lineages, bidirectional sequencing was performed to identify the correct lineage. New lineages were also sequenced in both directions, named following Bensch et al. ^27^ and deposited in GenBank (GenBank accession no PQ798937 - PQ798945) and in the MalAvi database ^27^. When the PCR result was positive but the sequence did not yield a clear result, for example, due to a mixed infection, the result was determined as “*Plasmodium* spp.”. We found 9 samples infected by *Haemoproteus* lineages, but we did not include them in the analysis, with the exception of the phylogenetic analyses, because mosquitoes are not competent vectors for *Haemoproteus* ^47,48^.

A phylogenetic tree was constructed using Molecular Evolutionary Genetics Analysis (MEGA) software (version 11.0.13; https://www.megasoftware.net) ^49^, including all the lineages found in the study. For those lineages with known morphospecies in the MalAvi database ^27^, the corresponding morphospecies were also included. We also added the new lineage sequences and those lineages with the higher proportion of matches in the MalAvi and GenBank. Lineage CB1 (*Leucocytozoon majoris*) was used as the outgroup (see Table S3). Multiple alignments were carried out using Clustal W in MEGA software (version 11.0.13; https://www.megasoftware.net) ^49^. The Tamura-Nei substitution model has the best model fit as tested with jModelTest 2.1.6 ^50^ in CIPRES ^51^, and it was used to build the phylogeny using the Maximum Likelihood algorithm. Node support was estimated by bootstrap analysis with 1000 replications.

### Environmental variables

We generated environmental datasets for the years 2021 and 2022 to understand the relationship between environmental variables and *Plasmodium* prevalence, richness and diversity in mosquitoes. At each sampling site, we defined a 2 km radius buffer around the latitude and longitude centroid of each mosquito trap based on the mean flight distance of *Culex* mosquitoes ^39^. We extracted environmental data corresponding to the sampling months (March to November) for the years 2021 and 2022 from different sources (see Table S5). With these extracted monthly data, we calculated the average of each environmental variable for each locality and year (2021 or 2022). We extracted data from TerraClimate dataset ^52^ at a ~ 4 km resolution including accumulated precipitation, minimum, maximum, and mean temperature, vapor pressure, and evapotranspiration. This dataset was selected as it provides a continuous, high-frequency, and standardized source of climatic information across our entire study region for the full study period, allowing us to capture the broad, meso-scale environmental gradients relevant to mosquito and parasite ecology. Additionally, we used a Digital Elevation Model (DEM) from ASTER v3 ^53^. Normalized Difference Vegetation Index (NDVI) and land surface temperature were extracted from MODIS ^54,55^. Finally, we assessed the percentage of land favorable for mosquito breeding by extracting information on land uses from the Andalusia Natural Heritage Information System (SIPNA in Spanish) ^56^. Then, we calculated the percentage of land favorable for mosquito breeding by adding the surfaces occupied by land uses that correspond to saltmarshes, lagoons, rice fields and greenhouses in relation to the total surface (see Table S6). All data were extracted with R version 4.4.0 using raster and terra packages.

### Statistical analysis

All statistical analyses were performed in R (version 4.4.0). The prevalence of *Plasmodium* was estimated for each locality, year and mosquito species. Prevalence was used instead of incidence (presence/absence) because mosquito pools were analyzed. Prevalence was estimated using the Maximum Likelihood (MLE) and considering the number of mosquitoes in each pool (1 to 50 individuals) with the PoolPrev function from the PoolTestR package ^57^. We also estimated the richness of *Plasmodium* lineages (S) from rarefaction curves, standardizing values to a fixed number of pools to account for differences in sampling effort between mosquito species, and the diversity of *Plasmodium* lineages (measured with the Shannon index: *H*′ = −∑^s^_i=1_=p_*i*_ log_2_(*p*_*i*_), where p_i_ is the proportional abundance of the *Plasmodium* lineages i in each locality and year) calculated using the vegan package ^58^ for each locality, year and mosquito species. We used linear mixed-effects models (LMM), using the lme4 package ^59^, to analyze differences in *Plasmodium* prevalence, *Plasmodium* lineage richness and *Plasmodium* lineage diversity between mosquito species, with locality and year as random intercepts effects.

The relationships between environmental variables and *Plasmodium* prevalence, lineage richness and diversity, were assessed using separate Linear Mixed-Effects Models (LMMs) for *Cx. pipiens* and *Cx. perexiguus* using the lme4 package ^59^. Locality and year were included as random intercepts. Model selection was performed using stepwise forward selection based on Akaike’s Information Criterion (AIC). Given the relatively high number of environmental predictors in relation to the number of localities/years analyzed that determine sample size to detect associations with environmental predictors, this approach was used to reduce the risk of overfitting the models and to identify a reduced set of candidate models that could be reliably estimated. In cases where competing models exhibited a ∆AIC ≤ 2, the model with the lower parameter count was preferred to balance model fit and parsimony. To assess potential collinearity among environmental predictors, we first computed pairwise Pearson correlation coefficients and visualized them using a heatmap (see Fig. S2). This exploratory analysis allowed us to identify strongly correlated variables prior to model fitting. Following this, we calculated variance inflation factors (VIFs) for all predictors included in each LMM. Predictors with VIF values higher than 4 were considered indicative of problematic collinearity ^60^ and were therefore not included together in the same model. In all the models, we included only those localities with data for at least five mosquito pools in the analyses. Consequently, we excluded two localities for *Cx. pipiens* (see Table S1) and seven for *Cx. perexiguus* (see Table S2). Additionally, for diversity analyses, localities with no parasite lineages identified were excluded from the analyses. This resulted in the exclusion of six localities for *Cx. pipiens* (see Table S1) and eight for *Cx. perexiguus* (see Table S2). Model fit was checked using Dharma diagnostics and examining the normal distribution of the residuals using qqplots. All factors in the models were standardized to enable the comparison of estimator values. A p-value below 0.05 was considered indicative of statistical significance.

To compare the composition of *Plasmodium* lineage communities across localities, years and mosquito species, we used correspondence analysis (CA) with FactoMineR package ^61^. Only the most abundant lineages (those with a total frequency greater than 5%) were considered for the analysis to reduce the impact of rare lineages. Then we tested the differences between the most abundant lineages in *Cx. pipiens* and *Cx. perexiguus* using the CA coordinates (see Table S7). An ANOVA was performed including locality and year as random factors in the model, across each dimension of the CA. In addition, we performed a non-metric multidimensional scaling (NMDS) analysis based on Bray–Curtis dissimilarity using the vegan package ^58^, which yielded patterns consistent with those obtained from the CA and is therefore presented in the supplementary material Figure S3.

Finally, we compared the similarity in *Plasmodium* community composition across mosquito species and assessed the potential effect of geographic distance in explaining differences between localities. We estimated β-diversity matrices (referring to the variation in lineage composition among localities within the same mosquito species) using pairwise dissimilarity based on Jaccard’s distance, which considers presence-absence data (i.e., whether each *Plasmodium* lineage is present or absent in a given locality), and with the vegan package ^58^. To test the sensitivity of our results to the influence of rare lineages, we also calculated β-diversity matrices using the Bray-Curtis dissimilarity metric, which incorporates abundance data (i.e., the number of positive mosquito pools for each *Plasmodium* lineage per locality within the same mosquito species; see Table S8 and Table S9). We used a Mantel test (vegan package ^58^) with 999 permutations to calculate the correlation between β-diversity matrices of *Plasmodium* communities of *Cx. pipiens* and *Cx. perexiguus*. Furthermore, we used a Mantel test to calculate the correlation between β-diversity matrices of *Plasmodium* communities of *Cx. pipiens* and *Cx. perexiguus* and the geographical distance between localities. Finally, we used partial Mantel tests to assess the correlation between β-diversity matrices of *Plasmodium* communities in *Cx. pipiens* and *Cx. perexiguus* and (i) geographic distance while controlling for environmental relatedness (based on extracted environmental variables), and (ii) environmental relatedness while controlling for geographic distance.

## Supplementary Information

Below is the link to the electronic supplementary material.

Supplementary Material 1

## Data Availability

Sequencing data files of new *Plasmodium* lineages have been deposited in the GenBank (accession numbers are PQ798937–PQ798945) and MalAvi databases and are publicly available as of the date of publication. Data reported in this paper for statistical analyses are provided in Supplementary Material Tables S1, S2, S8 and S9. The raw data and R code used in this study are available in Digital CSIC: https://doi.org/10.20350/digitalCSIC/18286. Environmental variables were extracted from public repositories, as listed in Supplementary Material Table S5.
