# Supplementary material for: Environmental factors affect the communities of avian Plasmodium in two species of Culex mosquitoes
Source: Sci Rep. 2026 May 5;16:20638. doi: 10.1038/s41598-026-51361-w (PMC13333799; doi:10.1038/s41598-026-51361-w)
Supplement: Supplementary file 1 — Supplementary Material 1 [file 41598_2026_51361_MOESM1_ESM.docx]

**SUPPLEMENTARY INFORMATION**

**Table S1.** Number of *Culex pipiens* pools analyzed and prevalence, richness (S) and diversity (H’) of *Plasmodium* for each locality and year.

| Locality name | Year | Nºpools | Prevalence | S | H’ |
| --- | --- | --- | --- | --- | --- |
| Los Álamos | 2021 | 17 | 0.029 | 2 | 0.562 |
| Los Álamos | 2022 | 28 | 0.013 | 4 | 1.386 |
| Corrales | 2021 | 14 | 0.021 | 1 | 0.000 |
| Corrales | 2022 | 31 | 0.017 | 3 | 1.040 |
| Casa de los Ingleses | 2021 | 14 | 0.000 | 0 | - |
| Casa de los Ingleses | 2022 | 15 | 0.000 | 0 | - |
| Salinas del Astur | 2021 | 2 | 0.000 | 0 | - |
| Salinas del Astur | 2022 | 8 | 0.061 | 4 | 0.895 |
| EDAR Moguer | 2021 | 13 | 0.096 | 2 | 0.693 |
| EDAR Moguer | 2022 | 29 | 0.093 | 4 | 0.895 |
| EDAR Huelva | 2021 | 24 | 0.000 | 0 | - |
| EDAR Huelva | 2022 | 89 | 0.013 | 4 | 0.895 |
| Granja Escuela | 2021 | 15 | 0.031 | 1 | 0.000 |
| Granja Escuela | 2022 | 12 | 0.101 | 2 | 0.693 |
| Gibraleón | 2021 | 8 | 0.204 | 2 | 0.693 |
| Gibraleón | 2022 | 7 | 0.281 | 3 | 1.040 |
| Celestino Mutis | 2021 | 40 | 0.028 | 6 | 1.594 |
| Celestino Mutis | 2022 | 74 | 0.023 | 8 | 1.620 |
| Palacio de Doñana | 2021 | 7 | 0.333 | 0 | - |
| Palacio de Doñana | 2022 | 24 | 0.020 | 4 | 0.895 |
| El Rocío | 2021 | 0 | 0.000 | 0 | - |
| El Rocío | 2022 | 0 | 0.000 | 0 | - |
| Palomares del Río | 2021 | 81 | 0.017 | 2 | 0.693 |
| Palomares del Río | 2022 | 77 | 0.046 | 4 | 0.895 |
| Dehesa de Abajo | 2021 | 59 | 0.018 | 1 | 0.000 |
| Dehesa de Abajo | 2022 | 62 | 0.040 | 9 | 1.965 |
| Cañada de los Pájaros | 2021 | 49 | 0.031 | 0 | - |
| Cañada de los Pájaros | 2022 | 47 | 0.020 | 4 | 0.895 |
| Puebla del Río | 2021 | 125 | 0.001 | 1 | 0.000 |
| Puebla del Río | 2022 | 143 | 0.003 | 5 | 1.468 |
| Coría del Río | 2021 | 61 | 0.016 | 1 | 0.000 |
| Coría del Río | 2022 | 54 | 0.024 | 2 | 0.562 |

**Table S2.** Number of *Culex perexiguus* pools analyzed and prevalence, richness (S) and diversity (H’) of *Plasmodium* for each locality and year.

| Locality name | Year | Nºpools | Prevalence | S | H’ |
| --- | --- | --- | --- | --- | --- |
| Los Álamos | 2021 | 2 | 0.000 | 0 | - |
| Los Álamos | 2022 | 3 | 0.000 | 0 | - |
| Corrales | 2021 | 1 | 0.000 | 0 | - |
| Corrales | 2022 | 6 | 0.000 | 0 | - |
| Casa de los Ingleses | 2021 | 0 | 0.000 | 0 | - |
| Casa de los Ingleses | 2022 | 0 | 0.000 | 0 | - |
| Salinas del Astur | 2021 | 2 | 0.000 | 0 | - |
| Salinas del Astur | 2022 | 0 | 0.000 | 0 | - |
| EDAR Moguer | 2021 | 2 | 0.000 | 0 | - |
| EDAR Moguer | 2022 | 2 | 0.000 | 0 | - |
| EDAR Huelva | 2021 | 1 | 0.000 | 0 | - |
| EDAR Huelva | 2022 | 9 | 0.007 | 1 | 0.000 |
| Granja Escuela | 2021 | 5 | 0.112 | 1 | 0.000 |
| Granja Escuela | 2022 | 4 | 0.126 | 1 | 0.000 |
| Gibraleón | 2021 | 8 | 0.159 | 2 | 0.693 |
| Gibraleón | 2022 | 5 | 0.269 | 2 | 0.693 |
| Celestino Mutis | 2021 | 9 | 0.087 | 1 | 0.000 |
| Celestino Mutis | 2022 | 6 | 0.414 | 1 | 0.000 |
| Palacio de Doñana | 2021 | 9 | 0.016 | 1 | 0.000 |
| Palacio de Doñana | 2022 | 17 | 0.000 | 0 | - |
| El Rocío | 2021 | 1 | 0.000 | 0 | - |
| El Rocío | 2022 | 2 | 0.000 | 0 | - |
| Palomares del Río | 2021 | 67 | 0.003 | 1 | 0.000 |
| Palomares del Río | 2022 | 129 | 0.017 | 9 | 1.447 |
| Dehesa de Abajo | 2021 | 199 | 0.010 | 9 | 1.822 |
| Dehesa de Abajo | 2022 | 346 | 0.017 | 18 | 1.908 |
| Cañada de los Pájaros | 2021 | 428 | 0.005 | 10 | 1.520 |
| Cañada de los Pájaros | 2022 | 497 | 0.010 | 23 | 2.265 |
| Puebla del Río | 2021 | 186 | 0.003 | 4 | 1.321 |
| Puebla del Río | 2022 | 242 | 0.009 | 10 | 1.587 |
| Coría del Río | 2021 | 75 | 0.003 | 1 | 0.000 |
| Coría del Río | 2022 | 127 | 0.006 | 5 | 1.334 |

**Table S3.** GenBank accession codes for the lineages used in the phylogenetic analyses including those newly identified in this study. Novel lineages are highlighted in bold.

| Lineage | Genus | Accession code | Reference |
| --- | --- | --- | --- |
| CB1 | *Leucocytozoon* | AY393804 | 1 |
| COLPAL01 | *Haemoproteus* | KJ488710 | 2 |
| STAL2 | *Haemoproteus* | EF607290 | 3 |
| STRTUR01 | *Haemoproteus* | KJ488784 | 2 |
| STRURA03 | *Haemoproteus* | KJ488826 | 2 |
| TYTAL6 | *Haemoproteus* | KU528634 | 4 |
| AFR106 | *Plasmodium* | KM056567 | 5 |
| AFTRU5 | *Plasmodium* | DQ847263 | 6 |
| BLUTI10 | *Plasmodium* | JQ434696 | 7 |
| CIAE01 | *Plasmodium* | EF607288 | 3 |
| COLL1 | *Plasmodium* | AY831747 | 8 |
| CXBIT01 | *Plasmodium* | AB733351 | 9 |
| CXPER01 | *Plasmodium* | HM179147 | 10 |
| CXPER04 | *Plasmodium* | PQ798942 | This study |
| CXPER05 | *Plasmodium* | PQ798943 | This study |
| CXPER06 | *Plasmodium* | PQ798944 | This study |
| CXPER07 | *Plasmodium* | PQ798945 | This study |
| CXPIP23 | *Plasmodium* | JF411405 | 11 |
| CXPIP33 | *Plasmodium* | LR990849 | NA |
| CXPIP35 | *Plasmodium* | PQ798937 | This study |
| CXPIP36 | *Plasmodium* | PQ798938 | This study |
| CXPIP37 | *Plasmodium* | PQ798939 | This study |
| CXPIP38 | *Plasmodium* | PQ798940 | This study |
| CXPIP39 | *Plasmodium* | PQ798941 | This study |
| DELURB4 | *Plasmodium* | EU154346 | 12 |
| DELURB5 | *Plasmodium* | EU154347 | 12 |
| DONANA02 | *Plasmodium* | JX458327 | 13 |
| DONANA04 | *Plasmodium* | JX975223 | 13 |
| GRW04 | *Plasmodium* | AF254975 | 14 |
| GRW11 | *Plasmodium* | AY831748 | 8 |
| LINN1 | *Plasmodium* | DQ847270 | 6 |
| PADOM01 | *Plasmodium* | DQ058611 | 15 |
| PADOM02 | *Plasmodium* | DQ058612 | 15 |
| PAGRI02 | *Plasmodium* | JX196865 | NA |
| SGS1 | *Plasmodium* | AF495571 | 16 |
| SGS2 | *Plasmodium* | JN661991 | 17 |
| SYAT05 | *Plasmodium* | DQ847271 | 6 |
| SYAT24 | *Plasmodium* | AY831749 | 8 |
| TUMER06 | *Plasmodium* | KJ488827 | 2 |
| TUPHI09 | *Plasmodium* | MT912308 | 18 |
| YWT4 | *Plasmodium* | DQ368395 | 19 |

**References**

1. Hellgren, O., Waldenström, J., Bensch, S. A new PCR assay for simultaneous studies of *Leucocytozoon, Plasmodium*, and *Haemoproteu*s from avian blood. J. Parasitol. 2004; 90: 797-802.

2. Drovetski, S. V., Aghayan, S. A., Mata, V. A., Lopes, R. J., Mode, N. A., Harvey, J. A., Voelker, G. Does the niche breadth or trade-off hypothesis explain the abundance–occupancy relationship in avian Haemosporidia?. Mol. Ecol*.* 2014; 23, 3322-3329.

3. Krone, O., Waldenström, J., Valkiūnas, G., Lessow, O., Müller, K., Iezhova, T. A., [Fickel](javascript:;), J., Bensch, S. Haemosporidian blood parasites in European birds of prey and owls. J. Parasitol. 2008; 94, 709-715.

4. Salakij, C., Pornpanom, P., Lertwatcharasarakul, P., Kasorndorkbua, C., Salakij, J. *Haemoproteus* in barn and collared scops owls from Thailand. J. Vet. Sci. 2018; 19: 280-289.

5. Lutz, H. L., Hochachka, W. M., Engel, J. I., Bell, J. A., Tkach, V. V., Bates, J. M., Hackett, S.J., Weckstein, J. D. Parasite prevalence corresponds to host life history in a diverse assemblage of Afrotropical birds and haemosporidian parasites. PloS ONE. 2015; 10.

6. Hellgren, O., Waldenström, J., Pérez-Tris, J., Szöll, E., Si, Ö., Hasselquist, D., Krizanauskiene, A., Ottosson, U., Bensch, S. Detecting shifts of transmission areas in avian blood parasites—a phylogenetic approach. Mol. Ecol. 2007; 16: 1281-1290.

7. Ferrer, E. S., García-Navas, V., Sanz, J. J., Ortego, J. Molecular characterization of avian malaria parasites in three Mediterranean blue tit (*Cyanistes caeruleus*) populations. Parasitol. Res. 2012; 111: 2137-2142.

8. Pérez-Tris, J., Bensch, S. Dispersal increases local transmission of avian malarial parasites. Ecol. Lett*.*2005; 8: 838-845.

9. Kim, K. S., Tsuda, Y. Avian *Plasmodium* lineages found in spot surveys of mosquitoes from 2007 to 2010 at Sakata wetland, Japan: do dominant lineages persist for multiple years?. Mol. Ecol. 2012; 21: 5374-5385.

10. Njabo, K. Y., Cornel, A. J., Bonneaud, C., Toffelmier, E., Sehgal, R. N. M., Valkiūnas, G., [Russell](https://onlinelibrary.wiley.com/authored-by/RUSSELL/A.+F.), A.F., Smith, T. B. Nonspecific patterns of vector, host and avian malaria parasite associations in a central African rainforest. Mol. Ecol. 2011; 20: 1049-1061.

11. Inci, A., Yildirim, A., Njabo, K. Y., Duzlu, O., Biskin, Z., Ciloglu, A. Detection and molecular characterization of avian *Plasmodium* from mosquitoes in central Turkey.  Vet. Parasitol. 2012; 188: 179-184.

12. Marzal, A., Bensch, S., Reviriego, M., Balbontin, J., De Lope, F. Effects of malaria double infection in birds: one plus one is not two. J. Evol. Biol. 2008; 21: 979-987.

13. Ferraguti, M., Martinez-de la Puente, J., Munoz, J., Roiz, D., Ruiz, S., Soriguer, R., Figuerola, J. Avian *Plasmodium* in *Culex* and *Ochlerotatus* mosquitoes from southern Spain: effects of season and host-feeding source on parasite dynamics. PloS ONE. 2013; 8: e66237.

14. Bensch, S., Stjernman, M., Hasselquist, D., Örjan, Ö., Hannson, B., Westerdahl, H., Pinheiro, R. T. Host specificity in avian blood parasites: a study of *Plasmodium* and *Haemoproteus* mitochondrial DNA amplified from birds. Proc. R. Soc. Lond. B Biol. Sci. 2000; 267: 1583-1589.

15. Bonneaud, C., Pérez-Tris, J., Federici, P., Chastel, O., Sorci, G. Major histocompatibility alleles associated with local resistance to malaria in a passerine. Evolution. 2006; 60: 383-389.

16. Waldenström, J., Bensch, S., Kiboi, S., Hasselquist, D., Ottosson, U. Cross-species infection of blood parasites between resident and migratory songbirds in Africa. Mol. Ecol. 2002; 11: 1545-1554.

17. Ishtiaq, F., Beadell, J. S., Warren, B. H., Fleischer, R. C. Diversity and distribution of avian haematozoan parasites in the western Indian Ocean region: a molecular survey. Parasitol. 2012; 139: 221-231.

18. Harl, J., Himmel, T., Valkiūnas, G., Ilgūnas, M., Bakonyi, T., Weissenböck, H. Geographic and host distribution of haemosporidian parasite lineages from birds of the family Turdidae. Malar. J. 2020; 19: 1-35.

19. Pérez-Tris, J., Hellgren, O., Križanauskienė, A., Waldenström, J., Secondi, J., Bonneaud, C., Fjeldså, J., Hasselquist, D., Bensch, S. Within-host speciation of malaria parasites. PLoS ONE. 2007; 2: e235.

**Table S4.** Localities sampled in this study. Number of BGs and CDCs per sampled site and periodicity of sampling are provided. In Casa de los Ingleses and Salinas del Astur BG’s were only used in 2021.

| Locality name | Locality code | Coordinates | Nº BG | Nº CDC | Nº times sampled |
| --- | --- | --- | --- | --- | --- |
| Los Álamos | 0 | 37°16'43.3"N 6°54'36.8"W | 3 | 1 | Once per month |
| Corrales | 1 | 37°16'39.0"N 6°59'16.4"W | 3 | 1 | Once per month |
| Casa de los Ingleses | 2 | 37°10'59.1"N 6°58'19.7"W | 3 | 1 | Once per month |
| Salinas del Astur | 3 | 37°12'18.0"N 6°59'33.4"W | 3 | 1 | Once per month |
| EDAR Moguer | 4 | 37°16'10.8"N 6°51'39.1"W | 3 | 1 | Once per month |
| EDAR Huelva | 5 | 37°14'31.6"N 6°55'41.5"W | 3 | 1 | Once per month |
| Granja Escuela | 6 | 37°19'28.4"N 6°47'47.8"W | 3 | 0 | Once per month |
| Gibraleón | 7 | 37°22'54.8"N 6°59'14.5"W | 3 | 0 | Once per month |
| Celestino Mutis | 8 | 37°12'20.5"N 6°55'24.0"W | 3 | 1 | Once per month |
| Palacio de Doñana | 9 | 36°59'19.2"N 6°26'34.6"W | 3 | 1 | Once per month |
| El Rocío | 10 | 37°07'40.1"N 6°28'48.6"W | 3 | 0 | Once per month |
| Palomares del Río | 11 | 37°18'58.3"N 6°03'28.0"W | 3 | 0 | Once per week |
| Dehesa de Abajo | 12 | 37°13'01.7"N 6°11'08.0"W | 3 | 0 | Once per week |
| Cañada de los Pájaros | 13 | 37°14'17.9"N 6°07'45.6"W | 3 | 0 | Once per week |
| Puebla del Río | 14 | 37°16'14.9"N 6°03'51.8"W | 3 | 0 | Once per week |
| Coria del Río | 15 | 37°17'02.5"N 6°03'51.9"W | 3 | 0 | Once per week |

**Table S5**. Description of the environmental variables used in the study. Source for each variable, spatial and temporal resolution, and units of measurement are reported for each variable.

| Variables | Description | Source | Spatial resolution | Temporal resolution | Units |
| --- | --- | --- | --- | --- | --- |
| Maximum temperature (Tmax) | The highest temperature recorded in a given month | Terraclimate | ~ 4km | 2021-2022 | ºC |
| Minimum temperature (Tmin) | The lowest air temperature recorded in a given month | Terraclimate | ~ 4km | 2021-2022 | ºC |
| Mean temperature (Tmean) | The average air temperature in a given month | Terraclimate | ~ 4km | 2021-2022 | ºC |
| Precipitation | Total amount of precipitation in a given month | Terraclimate | ~ 4km | 2021-2022 | mm |
| Actual Evapotranspiration | Water loss measurement from the Earth’s surface | Terraclimate | ~ 4km | 2021-2022 | mm |
| Normalized Difference Vegetation Index (NDVI) | The monthly Green vegetation coverage index | MODIS  [MOD13Q1](http://doi.org/10.5067/MODIS/mod13q1.006)  Version 6.1 | 250m | 2021-2022 | No units |
| Land Surface Temperature (LST) | Monthly average land surface temperature | MODIS  [MOD11A2 Version 6.1](https://doi.org/10.5067/MODIS/MOD11A2.061) | 1km | 2021-2022 | ºC |
| Vapor pressure | Monthly average of vapor pressure (pressure exserted by vapor) | Terraclimate | ~ 4km | 2021-2022 | kpa |
| DEM-Digital Elevation Model | Topographic surface of the Earth | ASTER v2 | 30 m | 2022 | m |
| Land Uses (LND) | Proportion of land-use categories within buffers (see Appendix D) | SIPNA | Shapefile-Polygon | 2022 | % |

**Table S6.** Land use categories considered, those considered more favorable for mosquito breeding are marked in bold.

| Land code | Land use |
| --- | --- |
| 1 | Mount |
| 2 | River/Sea |
| 3 | Saline |
| 4 | **Salt marshes** |
| 5 | **Rice field** |
| 6 | Pine forest |
| 7 | Industrial zone |
| 8 | **Lagoons** |
| 9 | Urban zone |
| 10 | **Greenhouses** |
| 11 | Mines |

**Table S7.** Coordinates obtained in the Correspondence Analysis (CA) on the *Plasmodium* lineages detected in *Culex pipiens* and *Culex perexiguus* mosquitoes in each study locality and year.

| Locality name | Year | Mosquito species | Coordinates  Dim 1 | Coordinates  Dim 2 |
| --- | --- | --- | --- | --- |
| Los Álamos | 2021 | *Culex pipiens* | 4.129 | 0.192 |
| Los Álamos | 2022 | *Culex pipiens* | -0.247 | -0.0583 |
| Corrales | 2022 | *Culex pipiens* | 4.129 | 0.192 |
| Salinas Astur | 2022 | *Culex pipiens* | 4.129 | 0.192 |
| EDAR Moguer | 2021 | *Culex pipiens* | -0.189 | -1.162 |
| EDAR Moguer | 2022 | *Culex pipiens* | -0.189 | -1.162 |
| EDAR Huelva | 2022 | *Culex pipiens* | 2.192 | -0.037 |
| Granja Escuela | 2021 | *Culex pipiens* | -0.189 | -1.162 |
| Granja Escuela | 2022 | *Culex pipiens* | -0.247 | -0.058 |
| Granja Escuela | 2021 | *Culex perexiguus* | -0.189 | -1.162 |
| Granja Escuela | 2022 | *Culex perexiguus* | 4.129 | 0.192 |
| Gibraleón | 2021 | *Culex pipiens* | -0.189 | -1.162 |
| Gibraleón | 2022 | *Culex pipiens* | -0.159 | 0.740 |
| Gibraleón | 2021 | *Culex perexiguus* | -0.306 | 1.046 |
| Gibraleón | 2022 | *Culex perexiguus* | -0.247 | -0.058 |
| Celestino Mutis | 2021 | *Culex pipiens* | 0.307 | -0.165 |
| Celestino Mutis | 2022 | *Culex pipiens* | 0.002 | -0.393 |
| Celestino Mutis | 2022 | *Culex perexiguus* | -0.189 | -1.162 |
| Palacio de Doñana | 2021 | *Culex perexiguus* | -0.306 | 1.046 |
| Palomares del Río | 2021 | *Culex pipiens* | -0.247 | -0.058 |
| Palomares del Río | 2022 | *Culex pipiens* | -0.220 | -0.725 |
| Palomares del Río | 2021 | *Culex perexiguus* | -0.189 | -1.162 |
| Palomares del Río | 2022 | *Culex perexiguus* | -0.159 | 0.387 |
| Dehesa de Abajo | 2021 | *Culex pipiens* | -0.189 | -1.162 |
| Dehesa de Abajo | 2022 | *Culex pipiens* | 0.219 | -0.299 |
| Dehesa de Abajo | 2021 | *Culex perexiguus* | -0.232 | -0.399 |
| Dehesa de Abajo | 2022 | *Culex perexiguus* | -0.146 | 0.046 |
| Cañada de los Pájaros | 2022 | *Culex pipiens* | -0.271 | 0.102 |
| Cañada de los Pájaros | 2021 | *Culex perexiguus* | -0.275 | 0.472 |
| Cañada de los Pájaros | 2022 | *Culex perexiguus* | -0.147 | -0.174 |
| Puebla del Río | 2021 | *Culex pipiens* | -0.189 | -1.162 |
| Puebla del Río | 2022 | *Culex pipiens* | -0.211 | -0.134 |
| Puebla del Río | 2021 | *Culex perexiguus* | -0.236 | -0.279 |
| Puebla del Río | 2022 | *Culex perexiguus* | -0.101 | 0.252 |
| Coría del Río | 2021 | *Culex pipiens* | -0.189 | -1.162 |
| Coría del Río | 2022 | *Culex pipiens* | -0.189 | -1.162 |
| Coría del Río | 2021 | *Culex perexiguus* | -0.306 | 1.046 |
| Coría del Río | 2022 | *Culex perexiguus* | -0.287 | 0.450 |

**Table S8**. Abundance of Plasmodium lineages across sampling locations for *Cx. pipiens*. Values indicate the number of positive mosquito pools detected for each lineage at each location. Each row represents a distinct Plasmodium lineage, and each column corresponds to a sampling location. Letters correspond to the localities: (a) EDAR Huelva, (b) Granja Escuela, (c) Gibraleón, (d) Celestino Mutis, (e) Palacio de Doñana, (f) Palomares del Río, (g) Dehesa de Abajo, (h) Cañada de los Pájaros, (i) Puebla del Río, (j) Coria del Río.

|  | a | b | c | d | e | f | g | h | i | j |
| --- | --- | --- | --- | --- | --- | --- | --- | --- | --- | --- |
| AFR106 | 0 | 0 | 0 | 0 | 0 | 0 | 0 | 0 | 0 | 0 |
| AFTRU5 | 2 | 0 | 1 | 0 | 0 | 0 | 1 | 1 | 1 | 0 |
| BLUTI10 | 0 | 0 | 0 | 0 | 0 | 0 | 0 | 0 | 0 | 0 |
| CIAE01 | 0 | 0 | 0 | 0 | 0 | 0 | 1 | 0 | 0 | 0 |
| COLL1 | 0 | 0 | 0 | 0 | 0 | 0 | 0 | 0 | 0 | 0 |
| CXBIT01 | 0 | 0 | 0 | 0 | 0 | 0 | 0 | 0 | 0 | 0 |
| CXPER01 | 1 | 0 | 0 | 3 | 0 | 1 | 0 | 0 | 0 | 0 |
| CXPER04 | 0 | 0 | 0 | 0 | 0 | 0 | 0 | 0 | 0 | 0 |
| CXPER05 | 0 | 0 | 0 | 0 | 0 | 0 | 0 | 0 | 0 | 0 |
| CXPER06 | 0 | 0 | 0 | 0 | 0 | 0 | 0 | 0 | 0 | 0 |
| CXPER07 | 0 | 0 | 0 | 0 | 0 | 0 | 0 | 0 | 0 | 0 |
| CXPIP23 | 0 | 0 | 0 | 1 | 0 | 0 | 1 | 1 | 0 | 0 |
| CXPIP33 | 3 | 0 | 1 | 0 | 2 | 0 | 0 | 0 | 2 | 0 |
| CXPIP35 | 0 | 0 | 0 | 0 | 0 | 0 | 0 | 1 | 0 | 0 |
| CXPIP36 | 0 | 0 | 0 | 0 | 0 | 1 | 1 | 3 | 0 | 0 |
| CXPIP37 | 0 | 0 | 0 | 1 | 0 | 0 | 0 | 0 | 0 | 0 |
| CXPIP38 | 0 | 0 | 1 | 0 | 0 | 0 | 0 | 0 | 0 | 0 |
| CXPIP39 | 0 | 0 | 0 | 1 | 0 | 0 | 0 | 0 | 0 | 0 |
| DELURB4 | 4 | 0 | 0 | 0 | 0 | 0 | 0 | 0 | 0 | 0 |
| DELURB5 | 0 | 0 | 0 | 2 | 0 | 0 | 0 | 0 | 0 | 0 |
| DONANA02 | 0 | 0 | 0 | 0 | 0 | 0 | 0 | 0 | 0 | 0 |
| DONANA04 | 0 | 0 | 0 | 0 | 0 | 0 | 1 | 0 | 0 | 0 |
| GRW04 | 1 | 0 | 0 | 0 | 0 | 0 | 0 | 2 | 0 | 1 |
| GRW11 | 1 | 0 | 0 | 0 | 0 | 0 | 0 | 0 | 0 | 0 |
| LINN1 | 2 | 1 | 1 | 9 | 0 | 3 | 2 | 1 | 3 | 0 |
| PADOM01 | 0 | 0 | 0 | 0 | 0 | 0 | 0 | 0 | 0 | 0 |
| PADOM02 | 0 | 0 | 0 | 0 | 0 | 0 | 0 | 0 | 0 | 0 |
| PAGRI02 | 0 | 0 | 0 | 0 | 0 | 0 | 0 | 0 | 0 | 0 |
| SGS1 | 11 | 0 | 0 | 2 | 0 | 0 | 1 | 0 | 0 | 0 |
| SGS2 | 0 | 0 | 0 | 0 | 0 | 0 | 0 | 0 | 0 | 0 |
| SYAT05 | 5 | 3 | 1 | 16 | 0 | 11 | 7 | 1 | 6 | 4 |
| SYAT24 | 0 | 0 | 0 | 2 | 0 | 0 | 1 | 0 | 0 | 0 |
| TUMER06 | 0 | 0 | 0 | 0 | 0 | 0 | 0 | 0 | 0 | 0 |
| TUPHI09 | 0 | 0 | 0 | 0 | 1 | 0 | 0 | 0 | 0 | 0 |
| YWT4 | 0 | 0 | 0 | 1 | 0 | 0 | 0 | 0 | 1 | 0 |

**Table S9**. Abundance of Plasmodium lineages across sampling locations for *Cx. perexiguus*. Values indicate the number of positive mosquito pools detected for each lineage at each location. Each row represents a distinct Plasmodium lineage, and each column corresponds to a sampling location. Letters correspond to the localities: (a) EDAR Huelva, (b) Granja Escuela, (c) Gibraleón, (d) Celestino Mutis, (e) Palacio de Doñana, (f) Palomares del Río, (g) Dehesa de Abajo, (h) Cañada de los Pájaros, (i) Puebla del Río, (j) Coria del Río.

|  | a | b | c | d | e | f | g | h | i | j |
| --- | --- | --- | --- | --- | --- | --- | --- | --- | --- | --- |
| AFR106 | 0 | 0 | 0 | 0 | 0 | 0 | 1 | 0 | 0 | 0 |
| AFTRU5 | 0 | 0 | 0 | 0 | 0 | 2 | 14 | 6 | 6 | 0 |
| BLUTI10 | 0 | 0 | 0 | 0 | 0 | 0 | 1 | 1 | 0 | 0 |
| CIAE01 | 0 | 0 | 0 | 0 | 0 | 0 | 0 | 0 | 0 | 0 |
| COLL1 | 0 | 0 | 0 | 0 | 0 | 0 | 0 | 2 | 1 | 0 |
| CXBIT01 | 0 | 0 | 0 | 0 | 0 | 0 | 1 | 0 | 0 | 0 |
| CXPER01 | 0 | 0 | 0 | 1 | 0 | 1 | 0 | 7 | 2 | 2 |
| CXPER04 | 0 | 0 | 0 | 0 | 0 | 0 | 0 | 1 | 0 | 0 |
| CXPER05 | 0 | 0 | 0 | 0 | 0 | 0 | 1 | 0 | 0 | 0 |
| CXPER06 | 0 | 0 | 0 | 0 | 0 | 0 | 0 | 2 | 0 | 0 |
| CXPER07 | 0 | 0 | 0 | 0 | 0 | 0 | 0 | 1 | 0 | 0 |
| CXPIP23 | 0 | 0 | 0 | 0 | 0 | 0 | 4 | 6 | 1 | 0 |
| CXPIP33 | 0 | 0 | 0 | 0 | 0 | 0 | 1 | 0 | 0 | 0 |
| CXPIP35 | 0 | 0 | 0 | 0 | 0 | 1 | 12 | 6 | 0 | 0 |
| CXPIP36 | 0 | 0 | 0 | 0 | 0 | 3 | 12 | 1 | 5 | 2 |
| CXPIP37 | 0 | 0 | 0 | 0 | 0 | 0 | 0 | 0 | 0 | 0 |
| CXPIP38 | 0 | 0 | 0 | 0 | 0 | 0 | 1 | 3 | 1 | 0 |
| CXPIP39 | 0 | 0 | 0 | 0 | 0 | 0 | 0 | 0 | 0 | 0 |
| DELURB4 | 0 | 0 | 1 | 0 | 0 | 0 | 0 | 0 | 0 | 0 |
| DELURB5 | 1 | 0 | 0 | 0 | 0 | 1 | 1 | 9 | 3 | 3 |
| DONANA02 | 0 | 0 | 0 | 0 | 0 | 0 | 2 | 3 | 0 | 0 |
| DONANA04 | 0 | 0 | 0 | 0 | 0 | 0 | 2 | 2 | 0 | 0 |
| GRW04 | 0 | 0 | 0 | 0 | 0 | 0 | 2 | 11 | 1 | 0 |
| GRW11 | 0 | 0 | 0 | 0 | 0 | 0 | 1 | 0 | 0 | 0 |
| LINN1 | 0 | 0 | 2 | 0 | 1 | 25 | 47 | 61 | 32 | 14 |
| PADOM01 | 0 | 0 | 0 | 0 | 0 | 0 | 0 | 1 | 0 | 0 |
| PADOM02 | 0 | 0 | 0 | 0 | 0 | 0 | 0 | 1 | 0 | 0 |
| PAGRI02 | 0 | 0 | 0 | 0 | 0 | 0 | 0 | 0 | 0 | 0 |
| SGS1 | 0 | 1 | 0 | 0 | 0 | 1 | 2 | 2 | 2 | 0 |
| SGS2 | 0 | 0 | 0 | 0 | 0 | 0 | 0 | 2 | 0 | 0 |
| SYAT05 | 0 | 1 | 1 | 1 | 0 | 11 | 53 | 57 | 2 | 4 |
| SYAT24 | 0 | 0 | 0 | 0 | 0 | 0 | 1 | 2 | 0 | 0 |
| TUMER06 | 0 | 0 | 0 | 0 | 0 | 2 | 1 | 1 | 0 | 0 |
| TUPHI09 | 0 | 0 | 0 | 0 | 0 | 0 | 0 | 0 | 0 | 0 |
| YWT4 | 0 | 0 | 0 | 0 | 0 | 0 | 1 | 0 | 0 | 0 |

**
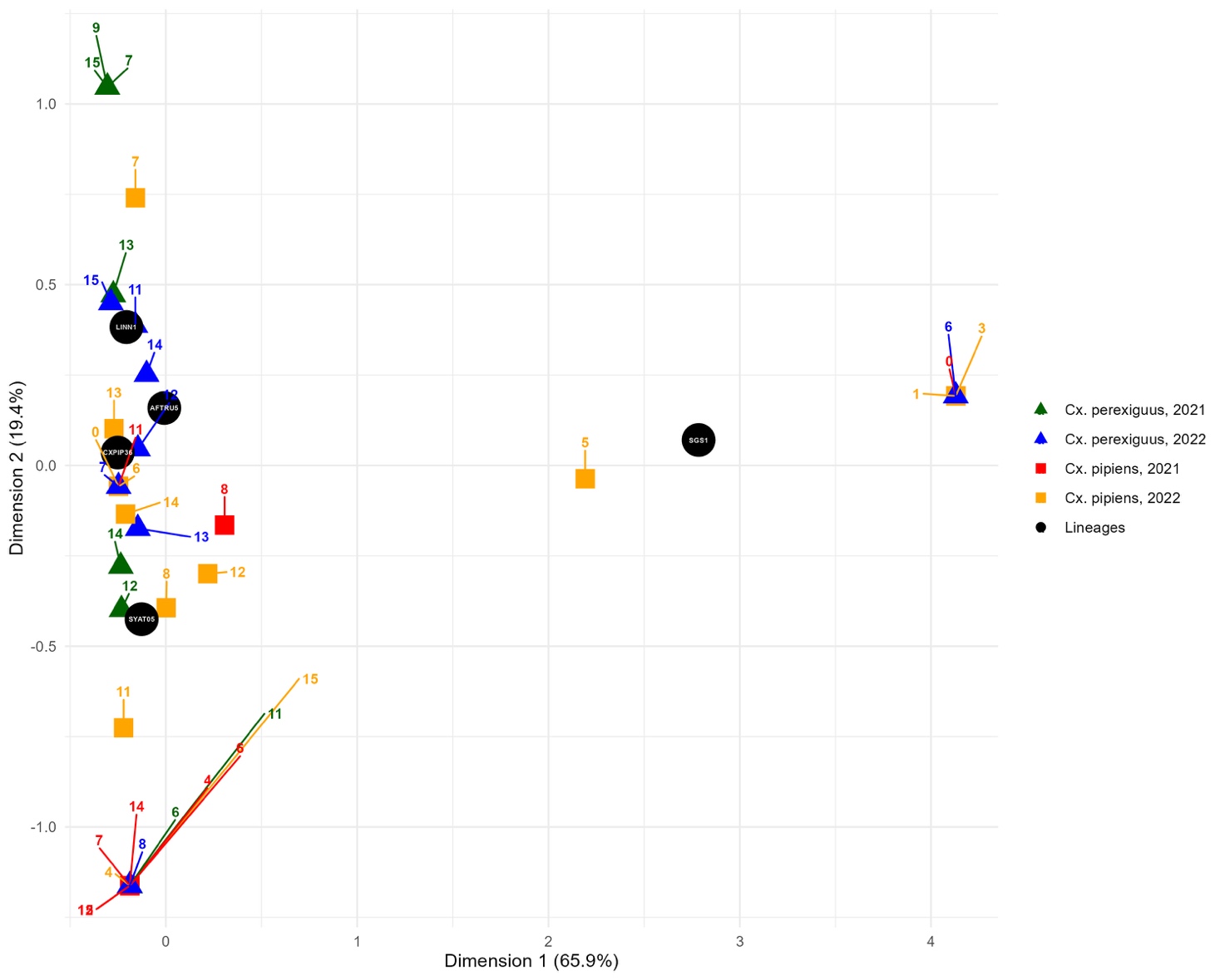
**

**Figure S1.** Correspondence analysis of the most abundant *Plasmodium* lineages found in *Culex pipiens* and *Culex perexiguus*. The plot highlights the distribution and relationship of different lineages across both vectors and years in the sampled localities. Numbers represent locality codes (see Table S4).


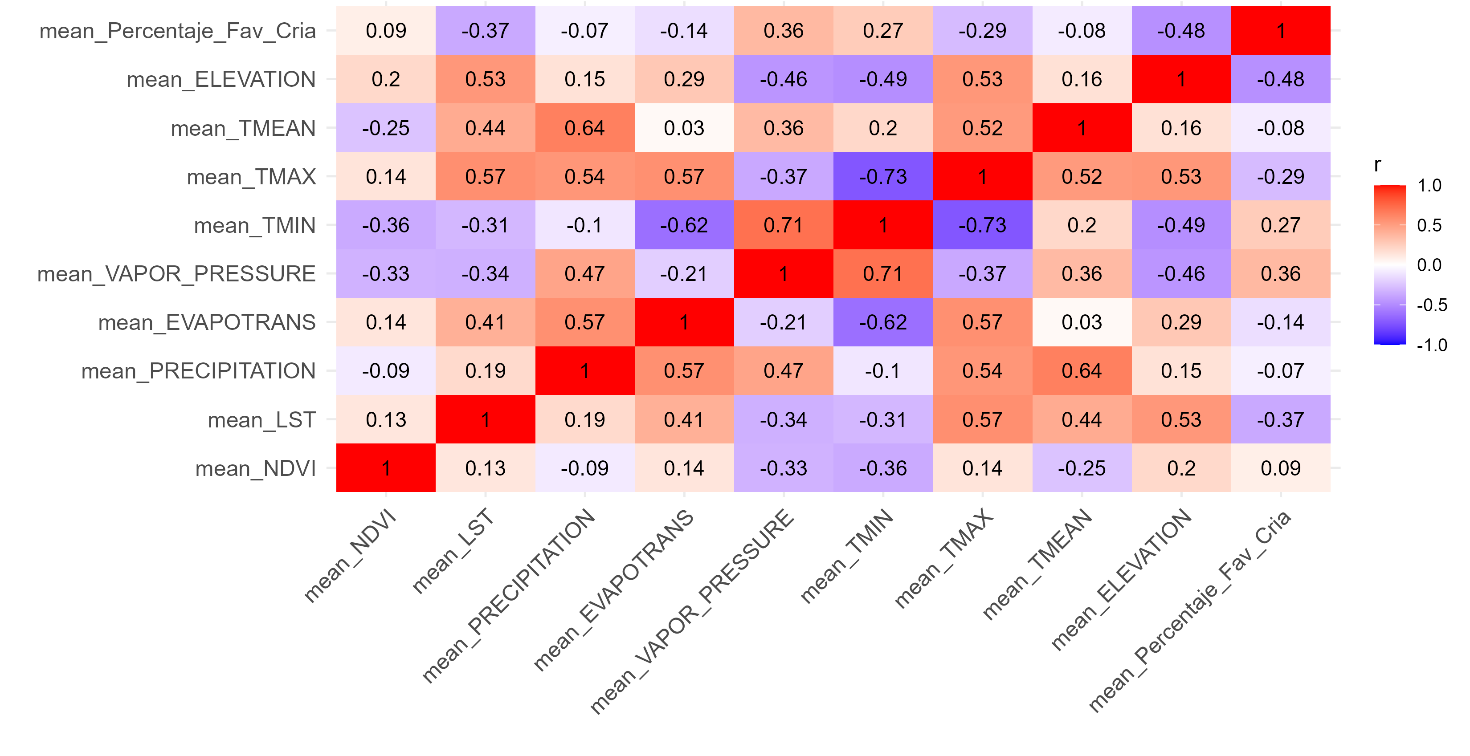


**Figure S2**. Heatmap showing pairwise correlations among environmental predictors used in the LMMs. Each cell displays the Pearson correlation coefficient (r) rounded to two decimal places. Blue indicates negative correlations, red indicates positive correlations, and white indicates near-zero correlations. Predictor abbreviations are defined as follows: mean_Percentage_Fav_Cria = annual mean percentage of land favorable for mosquito breeding; mean_ELEVATION = annual mean elevation; mean_TMEAN = annual mean mean temperature; mean_TMAX = annual mean maximum temperature; mean_TMIN = annual mean minimum temperature; mean_VAPOR_PRESSURE = annual mean vapor pressure; mean_EVAPOTRANS = annual mean evapotranspiration; mean_PRECIPITATION = annual mean precipitation; mean_LST = annual mean land surface temperature; and mean_NDVI = annual mean normalized difference vegetation index.


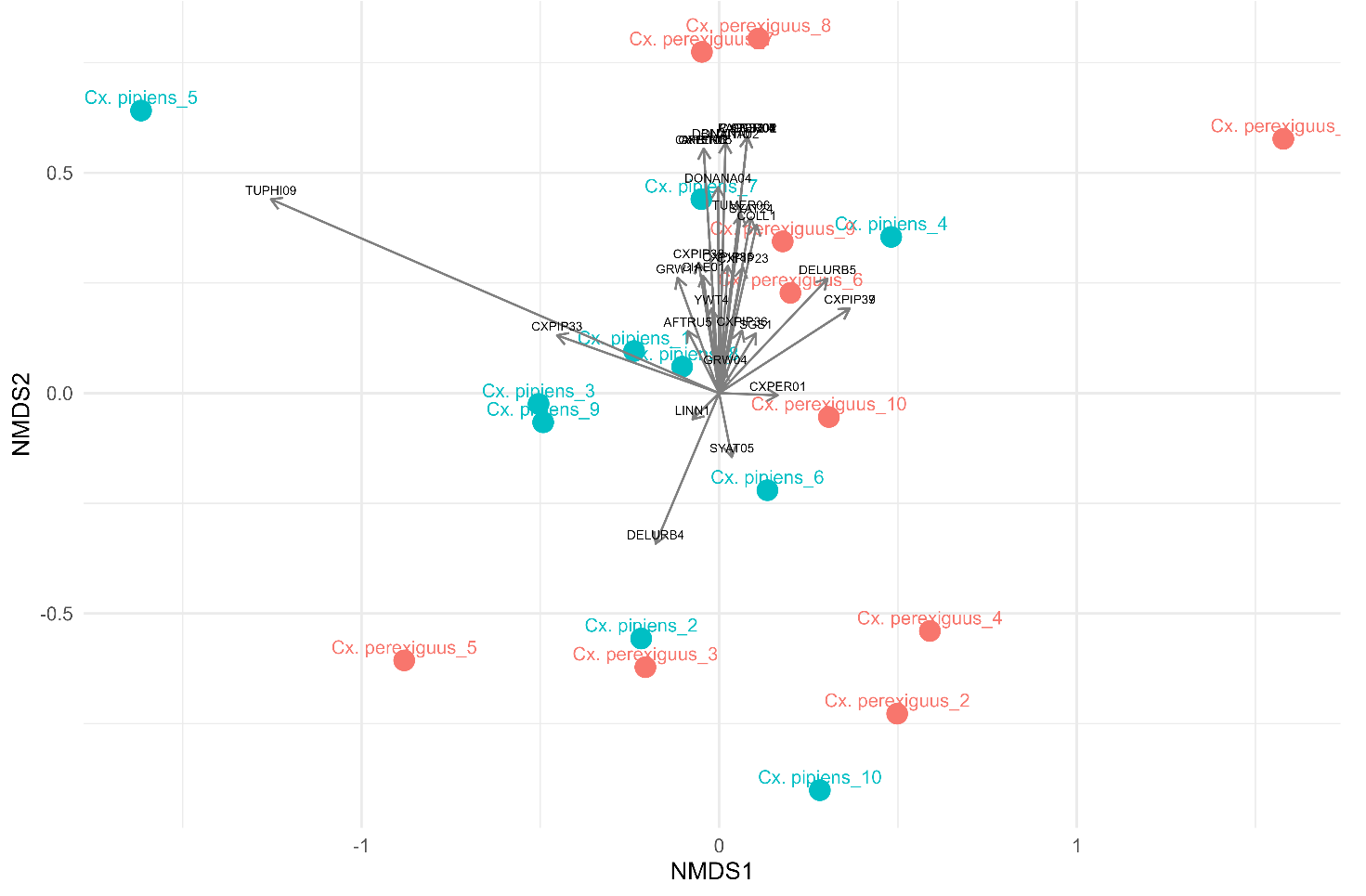


**Figure S3**. Non-metric multidimensional scaling (NMDS) plot based on Bray–Curtis dissimilarity, showing the ordination of *Cx. pipiens* (blue) and *Cx. perexiguus* (red) mosquito populations across different localities. Each point represents a population from a specific locality, labeled with its corresponding numbered code (see Table S4). Black arrows indicate *Plasmodium* lineages, with their orientation and length reflecting the strength and direction of their association with the mosquito populations.
